# Assessing perivascular spaces as a biomarker of Alzheimer’s disease in cognitively normal older adults

**DOI:** 10.1002/alz.095784

**Published:** 2025-01-09

**Authors:** Lauren Abigail Houchin, Jenna N. Adams, Soyun Kim, Lisa M. Taylor, Mithra Sathishkumar, Liv McMillan, Alyssa L. Harris, Niels Janssen, Michael A. Yassa

**Affiliations:** ^1^ University of California, Irvine, Irvine, CA USA; ^2^ Universidad de La Laguna, Santa Cruz de Tenerife Spain

## Abstract

**Background:**

Perivascular spaces (PVS), the small spaces surrounding vasculature in the brain which are critical for glymphatic clearance, are an emerging marker of cerebrovascular dysfunction. Abnormal expansion of PVS has been associated with Alzheimer’s disease (AD), and, therefore, may provide a measure of disease risk. Here, we investigate the relationship between PVS burden, memory performance, neurodegeneration, and biomarkers of AD pathology in community‐dwelling older adults without cognitive impairment.

**Method:**

We included 120 participants from the Biomarker Exploration in Aging, Cognition, and Neurodegeneration (BEACoN) study. PVS segmentation was conducted utilizing T1‐weighted MRI and an open‐source deep learning algorithm (the “SHIVA‐perivascular spaces (PVS)” software package), producing two global outcome measures: PVS volume and number of PVS clusters. PVS volumes were converted to a volume fraction using white matter and basal ganglia volumes for each participant. We then investigated the relationship between perivascular spaces and 1) delayed recall scores on the Rey Auditory Verbal Learning Test (RAVLT), 2) hippocampal volume, derived from Freesurfer segmentation of T1‐weighted images and corrected for intracranial volume, and 3) amyloid‐beta (Aβ) pathology, assessed using 18F‐florbetapir‐PET, and quantified continuously with global SUVR values and thresholded for amyloid‐positivity at >1.11. Associations with PVS were tested using correlations and t‐tests with age, sex, and education as covariates.

**Result:**

Global PVS count and volume were not associated with RAVLT delayed recall scores. Additionally, while Aβ+ status was not significantly associated with PVS count or volume, a t‐test of mean SUVR using a median split of PVS count was trending toward significance (p = 0.07). Global PVS count was negatively correlated (r = ‐.228, p = 0.015) with hippocampal volume. When age, sex, and education were controlled for, the result was less robust but trending toward significance (p = 0.09).

**Conclusion:**

Our results suggest that PVS may reflect early neurodegenerative changes and may be indicative of early AD pathological changes. Future efforts will examine longitudinal relationships and regional PVS values in regions associated with learning and memory, such as the medial temporal lobe.

